# Ironing out COPD: ferroptosis-driven immune dysregulation, metabolic rewiring, and precision therapeutic opportunities

**DOI:** 10.3389/fimmu.2026.1630969

**Published:** 2026-02-27

**Authors:** Feng-Xian Ni, Hui-Hui Chen, Ze-Bo Jiang, Dong-Hui Huang

**Affiliations:** Zhuhai Hospital of Integrated Traditional Chinese and Western Medicine, Zhuhai, Guangdong, China

**Keywords:** COPD, ferroptosis, immune-metabolic crosstalk, lipid peroxidation, mitochondrial dynamics, precision inhalation therapy

## Abstract

Chronic obstructive pulmonary disease (COPD) is a global health crisis driven by oxidative stress and immune dysregulation. Emerging evidence positions ferroptosis—an iron-dependent cell death driven by iron-catalyzed peroxidation of esterified polyunsaturated fatty acids (PUFAs) in membrane phospholipids—as a pivotal mediator of COPD pathogenesis. This review synthesizes cutting-edge insights into how cigarette smoke (CS) induces mitochondrial fission (via dynamin-related protein 1 (DRP1) phosphorylation) to exacerbate ferroptosis, potentially by enhancing lipid droplet (LD)-mitochondria contact sites and promoting lipid peroxidation in airway epithelial cells. This review further elucidates the complex and context-dependent role of nuclear factor erythroid 2-related factor 2 (Nrf2). While Nrf2 signaling is often suppressed globally in COPD lungs, its dysfunction in macrophages may paradoxically promote ferritinophagy-mediated iron retention through nuclear receptor coactivator 4 (NCOA4), overwhelming ferroprotein (FPN)-mediated iron export and unintentionally fueling ferroptosis. Clinically, plasma malondialdehyde (MDA)—a byproduct of lipid peroxidation—serving as a biomarker of oxidative stress severity, with elevated levels correlating with accelerated lung function decline in COPD patients. Therapeutically, promising targeted strategies are highlighted, such as inhaled exosomes loaded with liproxstatin-1, which can selectively inhibit pulmonary ferroptosis without inducing system immunosuppression. By bridging molecular mechanisms to therapeutic innovation, this review outlines a roadmap for precision medicine in COPD, focusing on the ferroptosis-immune axis to disrupt the self-perpetuating cycle of inflammation and tissue damage.

## 1 Introduction

Chronic obstructive pulmonary disease (COPD) is a highly prevalent, progressive respiratory disorder characterized by persistent airway inflammation, irreversible airflow limitation, and alveolar destruction (1, 2). As a leading cause of global morbidity and mortality, COPD imposes a substantial economic and healthcare burden (3–6). The primary risk factor for COPD is chronic exposure to noxious particles, particularly cigarette smoke (CS), environmental pollutants, and occupational dusts (7, 8). The pathogenesis of COPD has been historically attributed to a complex interplay between sustained oxidative stress and chronic inflammation, leading to repetitive tissue injury and aberrant repair. Oxidative stress, predominantly fueled by CS, depletes antioxidant defenses, causing direct cellular damage and activating a network of inflammatory signaling pathways. This is coupled with profound immune dysregulation, characterized by the recruitment and persistent activation of innate and adaptive immune cells—including macrophages, neutrophils, and T lymphocytes—that release a barrage of pro-inflammatory cytokines, proteases, and reactive oxygen species (ROS), thereby perpetuating a vicious cycle of inflammation, tissue destruction, and remodeling (9–11). In recent years, the exploration of regulated cell death pathways has provided transformative insights into the pathophysiology of chronic diseases. While the roles of apoptosis and necrosis in COPD have been extensively documented, ferroptosis—a novel, iron-dependent form of regulated cell death driven by lipid peroxidation—has emerged as a critical but underexplored contributor (12). Distinct from other cell death modalities, ferroptosis involves glutathione (GSH) depletion, inactivation of glutathione peroxidase 4 (GPX4), and the lethal accumulation of lipid peroxide. Distinct from other forms of cell death, ferroptosis involves glutathione depletion, compromised glutathione peroxidase 4 (GPX4) activity, and unrestrained lipid peroxide accumulation, governed by intricate metabolic and signaling networks encompassing iron homeostasis, lipid metabolism, and pathways such as p53, MAPK, and Nrf2 (13, 14).

Emerging evidence now positions ferroptosis not merely as a consequence but as a key amplifier of COPD pathology, particularly through its dynamic interplay with immune dysregulation (9, 15, 16). However, the precise molecular crosstalk between ferroptotic cell death and the immune landscape in the COPD lung remains a significant knowledge gap. This review aims to provide a comprehensive synthesis of current knowledge on the ferroptosis-immune axis in COPD. We will examine the molecular mechanisms of ferroptosis, its nuanced regulation within specific pulmonary cell types and organelles, its bidirectional relationship with immune activation, and the consequent therapeutic implications. Notably, this review aims to provide a comprehensive synthesis by: (1) integrating findings across cell biology, immunology, and metabolism to elucidate the ferroptosis-immune-metabolic network in COPD; (2) emphasizing underappreciated, organelle-specific regulatory mechanisms (e.g., mitochondrial fission, ferritinophagy, LD-mitochondria crosstalk) in pulmonary ferroptosis; and (3) translating mechanistic insights into a critical evaluation of emerging therapeutic strategies, including inhaled nanotherapeutics and biomarker-guided precision medicine. By doing so, we aim to provide a transformative framework for understanding COPD pathogenesis and inform the development of novel, targeted interventions.

## 2 Ferroptosis: a novel form of regulated cell death

Ferroptosis is a distinctive type of regulated cell death that significantly differs from apoptosis, necrosis, and autophagy. First identified in 2012, it is defined by iron-dependent lipid peroxidation and the accumulation of lipid peroxides (17, 18). Key features of ferroptosis include compromised plasma membrane integrity, mitochondrial dysfunction, and excessive ROS production (19). Recent research has uncovered complex molecular mechanisms underlying ferroptosis, with implications for diseases such as neurodegenerative disorders, cancer, and COPD (20).

### 2.1 Core machinery of ferroptosis

**Figure 1:**
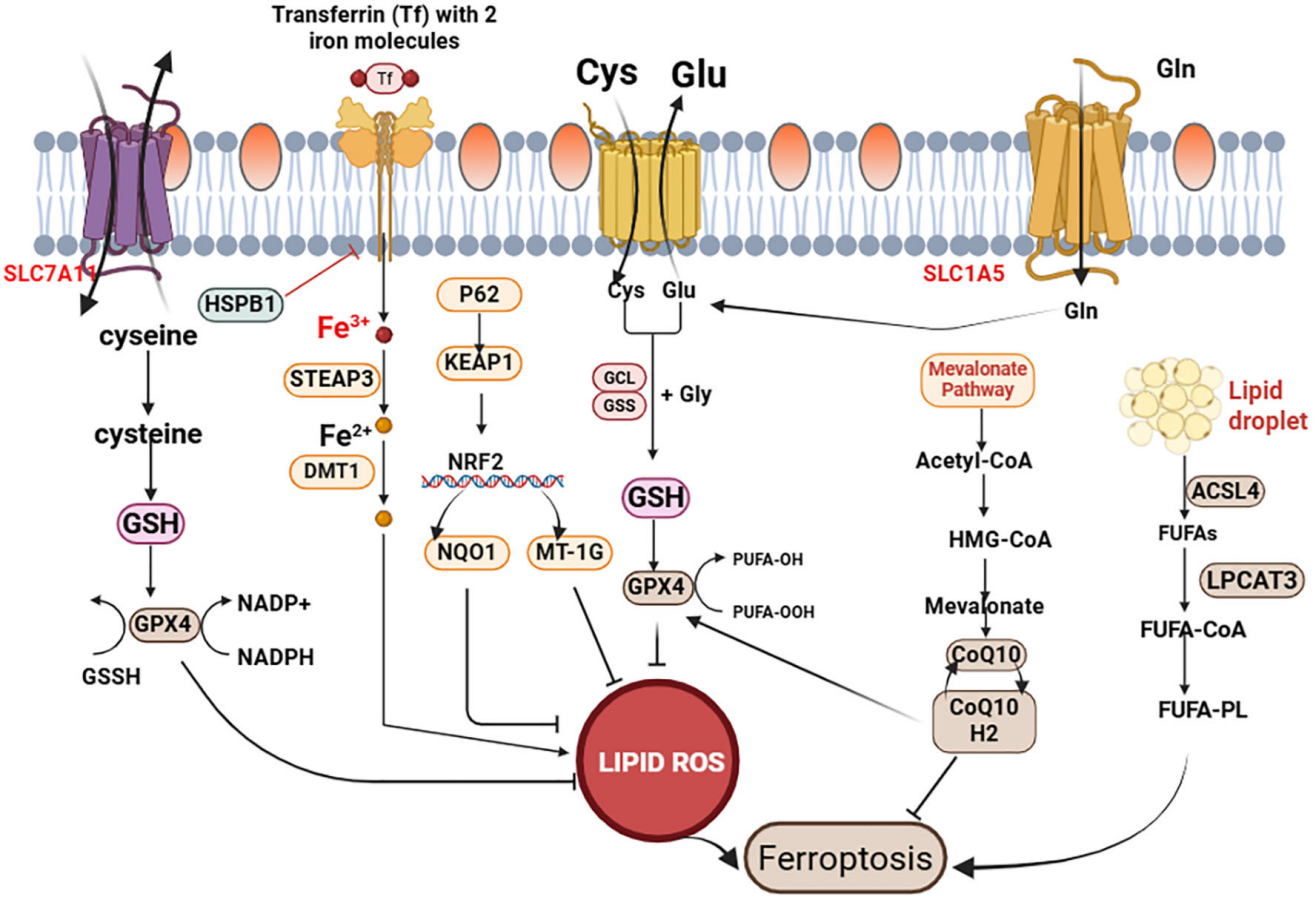
Mechanisms of the ferroptosis. Ferroptosis is regulated by three key systems: (1) GPX4, which reduces lipid hydroperoxides to prevent their accumulation; (2) System Xc^-^ (composed of SLC7A11 and SLC3A2), which imports cystine for GSH synthesis; (3) ACSL4, which promotes the integration of polyunsaturated fatty acids (PUFAs) into cell membranes, enhancing lipid peroxidation susceptibility. Mitochondrial dysfunction, iron overload, and ER stress further modulate ferroptosis sensitivity.

The regulation of ferroptosis involves several key components, including GPX4, the cystine/glutamate antiporter system (System Xc-, composed of SLC7A11 and SLC3A2 subunits) and acyl-CoA synthetase long-chain family member 4 (ACSL4) (17, 18, 21, 22) (**Figure 1**). Understanding the roles and interactions of these molecules is critical for deciphering ferroptosis pathways and their relevance to COPD (**Table 1**).

**Table 1:** Expression changes and functional outcomes of key ferroptosis-related molecules in COPD.

Gene/protein	Cell type	Functional outcome	Reference
GPX4↓	Airway epithelial cells	Reduced antioxidant defense, increased lipid peroxidation, and aggravated cell death	(75, 151, 152)
SLC7A11↓	Alveolar macrophages	Reduced cystine uptake, limited GSH synthesis, decreased antioxidant capacity, sensitivity to ferroptosis ↑	(153)
Nrf2↓	Airway epithelial cells	Downregulated antioxidant genes (e.g., HO-1, SLC7A11), promoting oxidative stress and ferroptosis	(75, 154)
ACSL4↑	Airway epithelial cells	Enhanced incorporation of PUFAs into membrane phospholipids, driving lipid peroxidation	(154)
LPCAT3↑	Airway epithelial cells	Increased membrane integration of oxidation-sensitive PUFAs	(155)
TfR1↑	Airway epithelial cells	Enhanced iron uptake, intracellular iron overload, and Fenton reaction-mediated lipid peroxidation	(156)
FTH1↓	Airway smooth muscle cells	Reduced iron storage capacity, elevated free iron levels, and promoted lipid peroxidation	(157)
FTL↓	Alveolar macrophages	Decreased ferritin light chain, increased iron release, and exacerbated oxidative stress	(135)
SLC40A1 (FPN) ↓	Endothelial cells	Impaired iron export, intracellular iron accumulation, and enhanced Fenton reaction	(158)
NCOA4↑	Alveolar macrophages	Enhanced ferritinophagy, increased iron release, and promoted ferroptosis	(159)
NOX4↑	macrophages	Increased ROS production and enhanced lipid peroxidation	(160)
FSP1↓	Fibroblasts	Impaired CoQ10-NAD(P)H pathway, reduced neutralization of lipid radicals	(161)
HO-1↓	Alveolar macrophages	Impaired antioxidant and anti-inflammatory functions, exacerbating ferroptosis-associated damage	(162)

**Table 1** summarizes the expression changes of key ferroptosis-related molecules in COPD, their cell-specific localization, and functional outcomes. All entries are supported by references relevant to COPD pathogenesis. ↑Upregulation; ↓downregulation.

#### 2.1.1 Glutathione peroxidase 4

Antioxidant defense systems, such as GPX4, play a critical role in protecting cells from ferroptosis (23, 24). As a selenoprotein, GPX4 catalyzes the reduction of lipid hydroperoxides to non-toxic lipid alcohols, preventing the accumulation of lipid peroxides (25). This enzymatic activity is essential for mitigating oxidative stress and maintaining cell viability, underscoring its importance in cellular homeostasis. In COPD, the pulmonary microenvironment is characterized by severe depletion of reduced glutathione (GSH) (146). Since GSH is a critical cofactor for GPX4, this depletion directly impairs GPX4 activity, rendering airway epithelial cells and alveolar macrophages highly susceptible to ferroptosis following oxidative insults such as CS exposure (26, 27). By converting lipid hydroperoxides into less harmful lipid alcohols, GPX4 helps maintain cellular membrane integrity and protects cells from the detrimental effects of lipid peroxidation.

#### 2.1.2 System Xc- (SLC7A11)

The cystine/glutamate antiporter System Xc- is a key regulator of cellular redox balance and antioxidant defense (28). Composed of the SLC7A11 (encoding the xCT subunit) and SLC3A2 subunits, it facilitates the import of cystine into cells in exchange for glutamate. Intracellular cystine is rapidly converted to cysteine—the rate-limiting precursor for GSH synthesis (29). GSH, in turn, scavenges ROS and maintains redox homeostasis, critical for protecting against ferroptosis (30). In the context of COPD, CS extract directly inhibits System Xc- activity, depleting intracellular cysteine and GSH pools (31). This disruption primes lung cells for ferroptosis by compromising their antioxidant capacity. By supplying cysteine for GSH synthesis, System Xc^-^ reinforces cellular defenses against oxidative damage, making it a key target for modulating ferroptosis in COPD.

#### 2.1.3 Acyl-CoA synthetase long-chain family member 4

ACSL4 is a pivotal enzyme in regulating lipid peroxidation and ferroptosis. It catalyzes the conversion of free long-chain fatty acids into acyl-CoA derivatives, facilitating their integration into cell membranes (32). This process, combined with the activity of lipoxygenases (LOXs) and other lipid-metabolizing enzymes, promotes lipid peroxidation and drives ferroptosis (29). ACSL4 preferentially activates polyunsaturated fatty acids (PUFAs), notably arachidonic and adrenic acids, which are highly susceptible to lipid peroxidation (33, 34). Notably, ACSL4 expression is upregulated in the airway epithelium of COPD patients and in experimental models of CS exposure (35). This upregulation suggests a direct link between CS-induced stress and the acquisition of a ferroptosis-sensitive lipid profile in lung cells. Inhibition of ACSL4 has been shown to protect cells from ferroptosis. For instance, Yingxi Wang et al. demonstrated that ACSL4 inhibition mitigated ferroptosis in tubular epithelial cells and reduced interstitial fibrosis (35).

#### 2.1.4 Iron-responsive element-binding protein 2

Other molecules involved in lipid and iron metabolism also regulate ferroptosis, including iron-responsive element-binding protein 2 (IRP2) (22). IRP2 is a transcription factor sensitive to intracellular iron levels, modulating the expression of proteins related to iron and lipid metabolism, thus influencing cellular susceptibility to ferroptosis (36). For example, Terzi et al. reported that the iron-sulfur cluster (ISC) could activate IRP2, enhancing sensitivity to ferroptosis through mechanisms independent of IRP1 and FBXL5 (37). Furthermore, other regulators of lipid metabolism, such as various lipases and lipid transporters, have been implicated in regulating lipid metabolism and ferroptosis (38). These components are significant in protecting cells from oxidative stress and lipid peroxidation and have been shown to sensitize cells to ferroptosis induction when depleted or inhibited.

### 2.2 Organelle-specific regulation of ferroptosis

**Figure 2:**
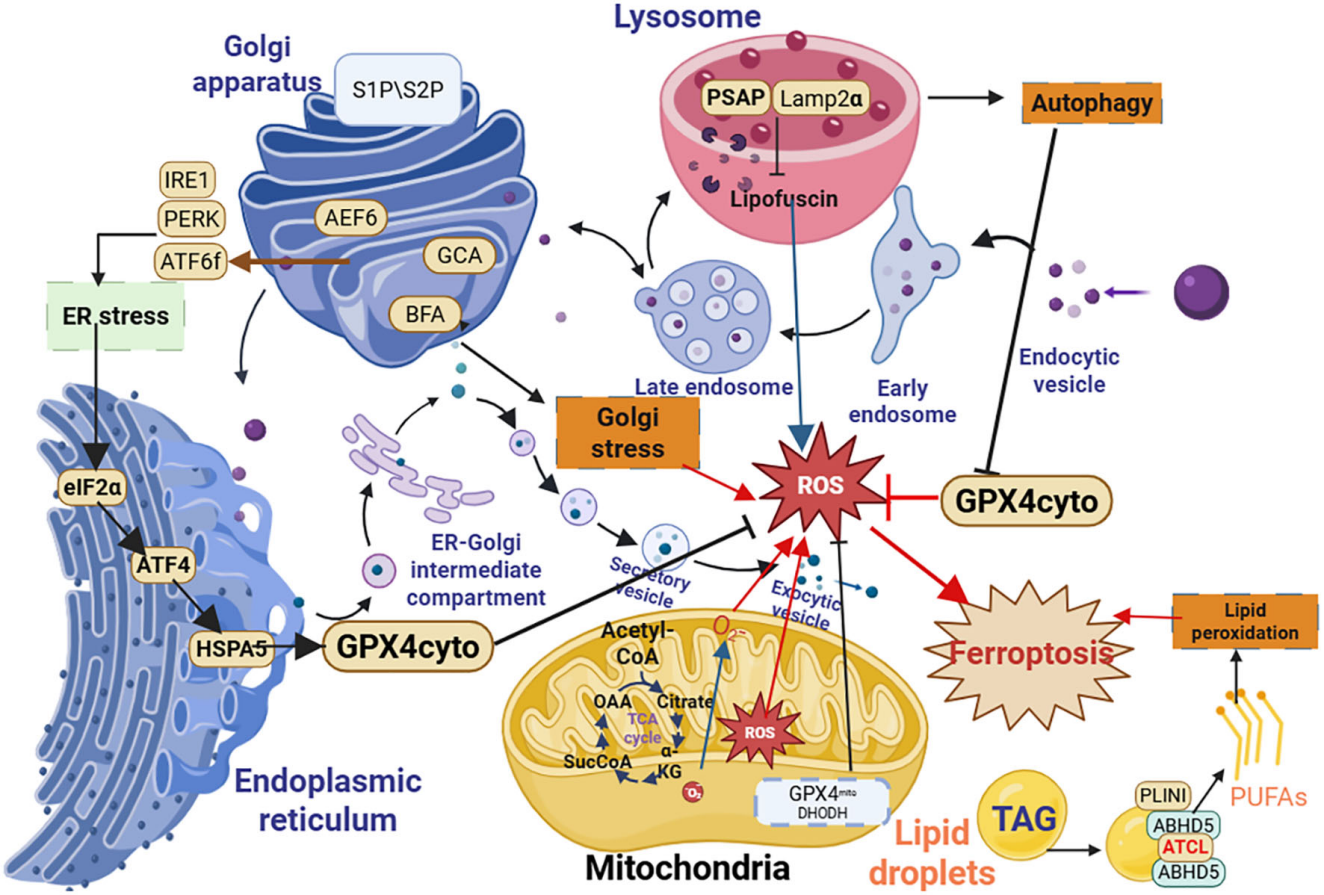
Organelle-specific regulation of ferroptosis. Mitochondria contribute to ferroptosis via DRP1-mediated fission and excessive ROS production. The ER regulates lipid synthesis (e.g., PUFAs) and System Xc^-^ activity. Lysosomes control iron release through ferritinophagy. LDs provide PUFAs for lipid peroxidation. Peroxisomes and the Golgi apparatus modulate lipid metabolism and antioxidant defense. In COPD, CS disrupts the function of these organelles, amplifying ferroptosis.

Organelle interactions are fundamental to the regulation of ferroptosis and include various cellular compartments such as mitochondria and the endoplasmic reticulum (ER), which work in conjunction with lipid metabolic pathways (39). These organelles critically modulate redox balance, lipid metabolism, and iron homeostasis during ferroptosis (22) (**Figure 2**).

#### 2.2.1 Mitochondria: regulatory mechanisms in ferroptosis

Mitochondria are critical for energy production, metabolism, and cell death regulation—including ferroptosis (40, 41). They maintain redox balance and iron homeostasis; dysfunction in these processes elevates oxidative stress, triggering ferroptosis (41). Mitochondria generate ATP via oxidative phosphorylation, a process that also produces ROS. Under physiological conditions, mitochondria balance ROS production with antioxidant defenses (42). However, stress (e.g., CS exposure) disrupts this balance, leading to excessive ROS accumulation (43, 44). Elevated ROS levels can trigger lipid peroxidation—an essential event in ferroptosis—by oxidizing PUFAs in cellular membranes, resulting in cellular damage and death. Mitochondrial dysfunction can exacerbate oxidative stress, heightening the risk of ferroptosis (45). Increased lipid peroxidation activates iron-dependent pathways that generate toxic lipid radicals, prompting cell demise. Iron is crucial for mitochondrial function, serving as a necessary component of mitochondrial proteins involved in the ETC and metabolic pathways. Mitochondria regulates both iron metabolism and storage; thus, maintaining optimal iron levels is essential for mitochondrial health. Dysregulation can lead to iron overload, catalyzing lipid peroxidation and oxidative stress, which promotes ferroptosis (46). Mitochondrial ferritin plays a protective role by sequestering excess iron and preventing oxidative damage (47). Peina Wang et al. reported that Mitochondrial ferritin attenuates cerebral ischemia/reperfusion injury by inhibiting ferroptosis (48). However, when mitochondrial ferritin is overwhelmed or dysfunctional, free iron levels may rise, catalyzing the Fenton reaction and exacerbating oxidative damage, further driving ferroptosis. For instance, Hypoxia inhibits ferritinophagy, increases mitochondrial ferritin, and protects from ferroptosis (49). Mitochondrial morphology, characterized by fission (division) and fusion (joining), significantly influences cellular susceptibility to ferroptosis. Fission can fragment mitochondria, making them more vulnerable to damage and dysfunction, while promoting the release of pro-apoptotic factors that increase cell death likelihood (50). Conversely, mitochondrial fusion maintains organelle integrity and functionality, countering oxidative stress effects (51). The balance between fission and fusion is regulated by specific proteins influenced by cellular signaling pathways in response to stressors. Dysregulation of these processes can tip the balance toward either cell survival or death, including ferroptosis; for example, inhibited fusion may enhance sensitivity to oxidative stress and promote ferroptotic cell death.

Mitochondria interact with other organelles to modulate ferroptosis. For example, mitochondrial dysfunction destabilizes lysosomes, amplifying oxidative stress (52). The ER-mitochondria interaction regulates calcium signaling; excessive ER calcium release disrupts mitochondrial function, promoting ferroptosis (53). Mitophagy—the selective degradation of damaged mitochondria—prevents ROS accumulation and ferroptosis (54); impairment of mitophagy leads to the accumulation of dysfunctional mitochondria, increasing oxidative damage and ferroptosis (55–57). Collectively, mitochondrial dynamics and function are critical regulators of ferroptosis in COPD.

#### 2.2.2 Endoplasmic reticulum: regulator of lipid metabolism and redox homeostasis

The ER maintains cellular homeostasis through protein synthesis, lipid metabolism, and calcium storage (58). Recent studies highlight its role in ferroptosis regulation, via ER stress responses, lipid biosynthesis, and iron metabolism (59). The ER is the primary site of lipid synthesis, producing phospholipids, cholesterol, and PUFAs—all of which influence membrane susceptibility to lipid peroxidation (60). ER stress—triggered by CS in COPD—escalates lipid peroxidation, activating ferroptosis pathways (61, 62). The ER also regulates redox homeostasis via the unfolded protein response (UPR). The UPR restores homeostasis by enhancing protein-folding capacity and reducing protein load; however, persistent ER stress activates cell death pathways, including ferroptosis (63). Prolonged ER stress increases oxidative stress and lipid peroxide accumulation, sensitizing cells to ferroptosis (64). For example, UPR activation upregulates enzymes involved in lipid metabolism, promoting lipid peroxidation. System Xc^-^—localized to the ER membrane—mediates cystine import for GSH synthesis (65). This impairment compromises antioxidant defense, increasing ferroptosis susceptibility. The ER also regulates iron metabolism by synthesizing and modifying ferritin; dysregulation of this process elevates free iron levels, amplifying lipid peroxidation (66). ER-mitochondria interactions are critical for ferroptosis regulation. Mitochondrial dysfunction increases ER stress, while ER calcium release disrupts mitochondrial function (67). In COPD, CS disrupts this crosstalk, exacerbating both ER stress and mitochondrial dysfunction, and amplifying ferroptosis (68). Targeting ER stress-e.g., via chemical chaperones or antioxidants—may inhibit ferroptosis, representing a potential therapeutic strategy for COPD (69).

#### 2.2.3 Lysosomes: controllers of iron homeostasis

Lysosomes regulate cellular debris degradation, biomolecule recycling, and iron metabolism—all of which influence ferroptosis (70, 71). Lysosomal membrane integrity is critical for maintaining cellular homeostasis; under ferroptotic conditions, lysosomal leakage releases hydrolytic enzymes and iron-rich contents into the cytosol, amplifying oxidative stress (72). Lysosomes mediate iron turnover via ferritinophagy—the selective degradation of ferritin (the primary iron storage protein) (73). When iron is needed, lysosomes degrade ferritin, releasing iron into the cytosol. Misregulation of this process leads to iron overload, which catalyzes the Fenton reaction and exacerbates lipid peroxidation. In COPD, CS exposure disrupts lysosomal function, increasing ferritinophagy and free iron levels (74, 75). Lysosomes interact with mitochondria and the ER to modulate ferroptosis. Mitochondrial ROS production induces lysosomal biogenesis (76), while impaired lysosomal function compromises mitochondrial health (64). The ER-lysosome interaction facilitates lipid transfer; disruption of this crosstalk disrupts lipid homeostasis, increasing lipid peroxide levels. Targeting lysosomal function-e.g., via lysosomal stabilizers or ferritinophagy inhibitors-may mitigate ferroptosis in COPD (16).

#### 2.2.4 Lipid droplets: sources of polyunsaturated fatty acids

Lipid droplets (LDs) are dynamic organelles serving as storage reservoirs for neutral lipids, playing pivotal roles in cellular energy homeostasis and lipid metabolism (77). Recent studies have revealed their significant involvement in regulating ferroptosis, characterized by iron-dependent lipid peroxidation. LDs influence ferroptosis primarily through lipid metabolism; they are essential for the storage and release of polyunsaturated fatty acids (PUFAs) (78). These fatty acids are particularly susceptible to oxidative damage, and their release from LDs can increase lipid peroxide levels within the cell, promoting ferroptotic cell death (78). Conversely, LDs can also store saturated fatty acids, which are less prone to oxidation, potentially safeguarding cells from ferroptotic insults. Thus, the balance between the types of lipids stored in LDs is a critical determinant of ferroptosis susceptibility. Additionally, LDs can modulate cellular redox status by sequestering ROS and providing a controlled environment for lipid peroxidation. Under conditions of increased cellular stress, LDs enhance the storage of toxic lipids that, upon mobilization, can trigger further oxidative stress and promote ferroptosis. Moreover, lipophagy—the selective degradation of lipid droplets—plays a significant role in these processes. When iron levels rise or oxidative stress increases, the autophagic degradation of LDs can release free fatty acids and accelerate lipid peroxidation, predisposing cells to ferroptosis.

#### 2.2.5 Peroxisomes: modulators of lipid oxidation and antioxidant defense

Peroxisomes regulate lipid metabolism and ROS detoxification, influencing ferroptosis (79). They mediate the β-oxidation of long-chain fatty acids, generating lipid-derived signaling molecules that alter membrane composition (80). Imbalances in fatty acid composition—e.g., increased PUFAs—increase ferroptosis susceptibility (41). Peroxisomes also degrade hydrogen peroxide via catalase, peroxisomal dysfunction leads to ROS accumulation, exacerbating lipid peroxidation. Peroxisomes interact with mitochondria to regulate metabolism and ROS dynamics (59). In COPD, CS disrupts peroxisomal function, reducing catalase activity and increasing PUFAs (111). This disruption amplifies ferroptosis, contributing to lung injury. Targeting peroxisomal function—e.g., via catalase activators or fatty acid oxidation modulators—may inhibit ferroptosis in COPD (112).

#### 2.2.6 Golgi apparatus: regulatory mechanisms in ferroptosis

The Golgi apparatus is a vital organelle involved in the post-translational modification, sorting, and trafficking of proteins and lipids within the cell (81). Recent discoveries indicate its significant role in regulating ferroptosis, characterized by iron-dependent lipid peroxidation. One mechanism by which the Golgi apparatus influences ferroptosis is through lipid modification, particularly phospholipid synthesis, which determines membrane integrity (82). By controlling lipid composition, the Golgi can affect cellular membranes’ susceptibility to oxidative damage. Additionally, it transports enzymes necessary for lipid metabolism, affecting the availability of PUFAs that are prone to oxidation (83). Furthermore, Golgi dysfunction can lead to the accumulation of misfolded proteins and increased oxidative stress, triggering signaling pathways that promote ferroptosis (84). Understanding the Golgi apparatus’s role in lipid and protein processing yields valuable insights into the mechanisms regulating ferroptosis and has implications for therapeutic strategies addressing diseases characterized by oxidative stress. In COPD, dysfunction in Golgi-mediated lipid trafficking could potentially contribute to ferroptotic sensitivity by altering the membrane composition of pulmonary cells.

Collectively, the dysfunction of key organelles—including mitochondria, endoplasmic reticulum, lysosomes, lipid droplets, peroxisomes, and the Golgi apparatus—synergistically amplifies ferroptosis susceptibility in COPD. Mitochondrial fission and ROS overproduction, ER stress-induced lipid peroxidation, lysosomal iron release via ferritinophagy, and altered lipid metabolism collectively disrupt cellular redox homeostasis. This organelle-centric vulnerability is further exacerbated by cigarette smoke, which perturbs inter-organelle communication and creates a pro-ferroptotic microenvironment. These subcellular alterations not only directly damage structural cells (e.g., airway epithelium) but also release damage-associated molecular patterns (DAMPs) and oxidized lipids that activate and recruit immune cells (85). Thus, the stage is set for a vicious cycle wherein organelle-driven ferroptosis and immune cell-mediated inflammation mutually reinforce each other, driving COPD progression. This interplay forms the foundation for exploring the role of immune cells in ferroptosis regulation, as detailed in the following section.

## 3 Immune cells in COPD

**Figure 3:**
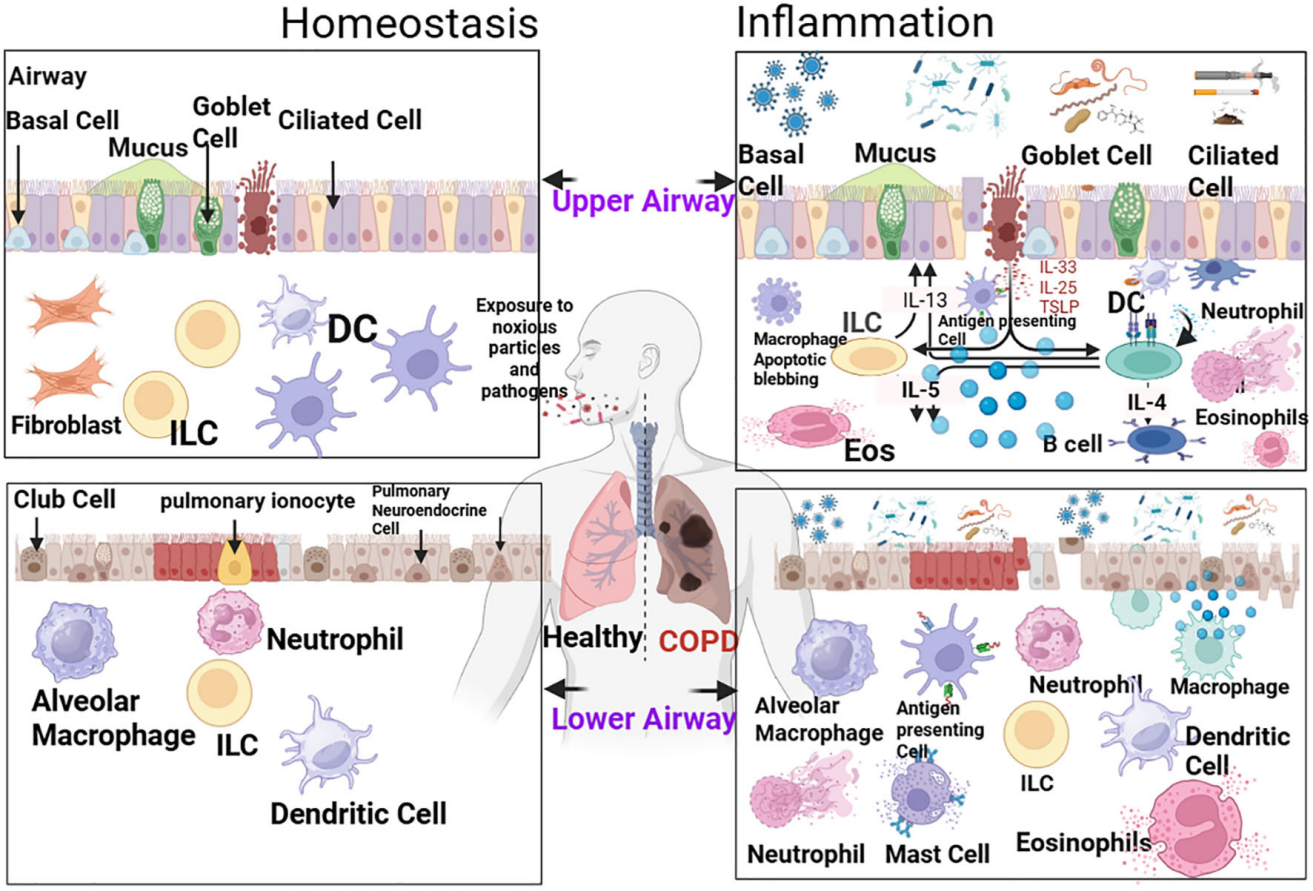
Provides a comprehensive schematic of the immune landscape in COPD pathogenesis, highlighting the interplay with ferroptosis. The diagram delineates three core components: (1) Pro-inflammatory drivers (M1 macrophages, Th17 cells, neutrophils) which, upon activation by cigarette smoke (CS), secrete cytokines (e.g., TNF-α, IL-6, IL-17), ROS, and proteases. These factors not only cause direct tissue damage but also promote ferroptosis in lung structural cells (airway epithelium, alveoli) by depleting antioxidants (GSH, GPX4) and enhancing lipid peroxidation. (2) Anti-inflammatory resolvers (M2 macrophages, Tregs) which secrete anti-inflammatory cytokines (IL-10, TGF-β) and support antioxidant defenses to inhibit inflammation and ferroptosis; however, their function is impaired in COPD. (3) Immune checkpoints (PD-1/PD-L1, TIM-3) which modulate T cell activity, with dysregulation contributing to T cell exhaustion and sustained inflammation. Crucially, the figure illustrates the feedback loop: ferroptotic cells release DAMPs and oxidized lipids (e.g., 4-HNE), which further activate pro-inflammatory immune cells, perpetuating the cycle. This visual model encapsulates the imbalance favoring pro-inflammatory and pro-ferroptotic signals in the COPD microenvironment.

Immune cells are integral to COPD pathogenesis, driving chronic inflammation, tissue damage, and airway remodeling. Key immune cells—including macrophages, neutrophils, T cells, and regulatory T cells (Tregs)—are recruited to the lungs in response to CS and other noxious stimuli (120) (**Figure 3**). These cells secrete pro-inflammatory cytokines, chemokines, and ROS, amplifying inflammation and activating ferroptosis. Emerging evidence indicates that ferroptosis modulates immune cell function, while immune cells regulate ferroptosis—creating a self-perpetuating cycle of inflammation and tissue damage. This section categorizes immune cells into pro-inflammatory drivers, anti-inflammatory resolvers, and immune checkpoints, and discusses their interactions with ferroptosis.

### 3.1 Pro-inflammatory drivers

Pro-inflammatory immune cells—including M1 macrophages, T helper 17 (Th17) cells, and neutrophils—drive inflammation and tissue damage in COPD. These cells enhance oxidative stress and ferroptosis, exacerbating disease progression (86).

#### 3.1.1 M1 macrophages

Macrophages are abundant in lung tissue and exist in two primary phenotypes: M1 (pro-inflammatory) and M2 (anti-inflammatory). In COPD, there is a shift toward M1 phenotype (121). M1 macrophages are activated by CS, bacterial products, and DAMPs, and secrete pro-inflammatory cytokines such as tumor necrosis factor-alpha (TNF-α) and interleukin-6 (IL-6) (87). These cytokines recruit additional immune cells, amplifying inflammation and tissue damage. M1 macrophages exhibit dysregulated iron metabolism, with increased expression of hepcidin (Hamp) and ferritin heavy/light chains (FTH/FTL), and reduced expression of ferroportin (FPN) and IRP1/2 (88). This dysregulation increases iron storage, promoting M1 polarization (89). Iron overload enhances glycolysis—supporting the M1 phenotype-and increases ROS production and p53 acetylation, further driving M1 polarization. M1 macrophages also promote ferroptosis in neighboring structural cells (e.g., airway epithelial cells) by secreting cytokines that downregulate GPX4 and SLC7A11 (90). This crosstalk creates a self-perpetuating cycle: inflammation induces ferroptosis, while ferroptotic cells release DAMPs that activate more M1 macrophages (91, 92). In COPD, M1 macrophage function is dysregulated via multiple pathways. The nuclear factor-kappa B (NF-κB) pathway-critical for inflammation—is hyperactivated in M1 macrophages, leading to sustained cytokine production (93, 94). The peroxisome proliferator-activated receptor gamma (PPARγ) pathway-which regulates macrophage polarization—is suppressed, further favoring M1 polarization (95, 96). M1 macrophages also contribute to airway remodeling by releasing proteases (e.g., matrix metalloproteinases) and growth factors, exacerbating airflow limitation (97). Targeting M1 macrophages—e.g., via NF-κB inhibitors or PPARγ agonists—may reduce inflammation and ferroptosis in COPD (98).

#### 3.1.2 Th17 cells

Th17 cells—a subset of CD4^+^ T cells that secrete IL-17 and interferon-gamma (IFN-γ)-play a critical role in COPD inflammation (99). Th17 cells are recruited to the lungs in response to CS, where they activate macrophages and neutrophils (100). IL-17 enhances neutrophil recruitment and ROS production, amplifying oxidative stress and ferroptosis (101). In COPD, Th17 cell numbers are increased, and their activity correlates with disease severity (102). Th17 cells promote ferroptosis by secreting cytokines that disrupt antioxidant defenses. For example, IL-17 downregulates SLC7A11 expression, reducing cystine uptake and GSH synthesis (103). This impairment increases lipid peroxidation and ferroptosis in lung structural cells. Th17 cells also activate neutrophils, which release ROS and proteases, further exacerbating ferroptosis. Targeting Th17 cells—e.g., via IL-17 inhibitors—may mitigate inflammation and ferroptosis in COPD.

#### 3.1.3 Neutrophils

Neutrophils are the most abundant granulocytes and serve as first responders in COPD (104). They are recruited to the lungs in response to cytokines from M1 macrophages and Th17 cells. Activated neutrophils release ROS, proteases (e.g., neutrophil elastase), and neutrophil extracellular traps (NETs), contributing to tissue damage and mucus hypersecretion (105). Neutrophilic inflammation is a hallmark of COPD exacerbations, worsening symptoms and lung function (106). Neutrophils directly induce ferroptosis by releasing excessive ROS, overwhelming cellular antioxidant defenses (107). They also secrete LOXs, which catalyze PUFA oxidation and lipid peroxidation. Conversely, ferroptotic cells release DAMPs that activate neutrophils, amplifying inflammation (108). In COPD, neutrophil function is dysregulated: CS impairs neutrophil apoptosis, prolonging their survival and inflammation. Neutrophils also exhibit increased NET formation, which contributes to tissue damage and ferroptosis (109). Targeting neutrophils—e.g., via neutrophil elastase inhibitors or NET inhibitors—may reduce inflammation and ferroptosis in COPD. For example, sivelestat (a neutrophil elastase inhibitor) has been shown to reduce lung injury in COPD models (110). However, further research is needed to evaluate the efficacy of these strategies in clinical settings.

### 3.2 Anti-inflammatory resolvers

Anti-inflammatory immune cells—including M2 macrophages and Tregs—resolve inflammation and promote tissue repair in COPD. These cells inhibit ferroptosis, creating a protective microenvironment. However, their function is impaired in COPD, leading to persistent inflammation and ferroptosis (111).

#### 3.2.1 M2 macrophages

M2 macrophages are recognized for their anti-inflammatory properties and their capacity to facilitate tissue repair. They facilitate wound healing by secreting anti-inflammatory cytokines and growth factors that help resolve inflammation after an acute immune response. However, in the context of chronic COPD, the function of M2 macrophages may be impaired, leading to an imbalance with M1 macrophages (112). This impairment leads to persistent inflammation and ongoing tissue damage, ultimately affecting ferroptosis regulation. Enhancing M2 macrophage activity could help mitigate chronic inflammation and ferroptosis responses, creating a more favorable environment for repair. Promoting M2 polarization or functionality could potentially reduce the incidence of ferroptosis in lung tissue, thereby alleviating damage (113, 114).

Modulating macrophage polarization, inhibiting pro-inflammatory signaling pathways, and promoting tissue repair mechanisms are potential approaches for attenuating inflammation, reducing tissue damage, and improving lung function in individuals with COPD. Pharmacological agents, such as corticosteroids, PPARγ agonists, and NF-κB inhibitors, have been investigated for their ability to modulate macrophage function and polarization in COPD (115). In addition to pharmacological interventions, cell-based therapies, such as macrophage transplantation or mesenchymal stem cell therapy, hold promises for modulating the inflammatory response and promoting tissue repair in COPD. These approaches aim to restore the balance between pro-inflammatory and anti-inflammatory macrophages, enhance the resolution of inflammation, and support lung regeneration. Further research is needed to optimize the efficacy and safety of these therapeutic strategies and to translate preclinical findings into clinical applications for individuals with COPD (116).

#### 3.2.2 Regulatory T cells

Tregs maintain immune homeostasis suppressing excessive inflammation (117). They secrete anti-inflammatory cytokines (e.g., IL-10, TGF-β) and inhibit pro-inflammatory cell activation (118). In COPD, Treg function is compromised, leading to uncontrolled inflammation (119). Treg depletion increases Th17 cell activity and neutrophil recruitment, amplifying ferroptosis. Tregs regulate ferroptosis by maintaining redox balance. They secrete GSH precursors (e.g., cysteine), enhancing antioxidant defenses in neighboring cells (120). Tregs also inhibit ROS production by pro-inflammatory cells, reducing oxidative stress and ferroptosis (121). Enhancing Treg function—e.g., via IL-2 administration or Treg adoptive transfer—may mitigate inflammation and ferroptosis in COPD (122). For example, IL-2 treatment has been shown to increase Treg numbers and reduce inflammation in COPD models (123).

### 3.3 Immune checkpoints in COPD

Immune responses in COPD are modulated by immune checkpoints that can either inhibit or enhance inflammation (124). Two significant checkpoints are the programmed death-1 (PD-1)/programmed death-ligand 1 (PD-L1) pathway and T cell immunoglobulin and mucin domain-containing protein 3 (TIM-3). Exploring their relationship with ferroptosis offers new insights into their potential as therapeutic targets for COPD (125).

#### 3.3.1 PD-1/PD-L1 pathway

The PD-1/PD-L1 axis is critical for regulating immune responses during chronic inflammation characteristic of COPD (126). PD-L1 expression on antigen-presenting cells inhibits T cell activation, contributing to immune tolerance or exhaustion. In COPD, this inhibition poses challenges since robust immune responses against damaging environmental factors are crucial. Notably, the PD-1/PD-L1 axis may influence ferroptosis; suppressed T cell activity can hinder the clearance of damaged cells, fostering an inflammatory environment rich in oxidative stress that triggers ferroptosis in surrounding lung tissue (127). Targeting the PD-1/PD-L1 pathway could potentially restore T cell functionality, enhance immune responses, and ameliorate ferroptosis.

#### 3.3.2 TIM-3 as a potential target

TIM-3 is another checkpoint that negatively regulates T cell responses. Its upregulation in chronic inflammatory conditions, such as COPD, may contribute to T cell exhaustion and ineffective immune responses (128–130). Recent studies suggest that TIM-3 may interact with ferroptosis signaling pathways, as chronic inflammation can induce senescence and ferroptosis in T cells, further compromising the immune response (131, 132). Developing therapeutic strategies to inhibit TIM-3 signaling could restore T cell activity and enhance immune efficacy against the inflammation and tissue damage characteristic of COPD (133). A comprehensive understanding of immune cell interactions—considering pro-inflammatory mediators, anti-inflammatory resolvers, immune checkpoints, and their relationships with ferroptosis—provides valuable insights into the underlying mechanisms of COPD. By addressing ferroptosis in both pro-inflammatory and anti-inflammatory contexts, new therapeutic strategies may be developed. Continued research into these pathways is essential for informing targeted therapies aimed at improving clinical outcomes and quality of life for individuals affected by this debilitating disease. Targeting both inflammation and ferroptosis could yield a multifaceted treatment approach that mitigates lung damage and promotes healing in COPD patients.

The distinct yet interconnected roles of immune cells—from the pro-ferroptotic actions of M1 macrophages, neutrophils, and Th17 cells to the protective efforts of M2 macrophages and Tregs—collectively establish a complex immunological landscape in COPD. However, these cell-specific effects do not occur in isolation. Instead, they form a dynamic, self-perpetuating network of crosstalk with ferroptotic lung cells, wherein immune-derived signals amplify lipid peroxidation, and ferroptosis-derived DAMPs further fuel inflammation. This intricate reciprocity underscores the necessity to move beyond a cell-centric view and toward a holistic understanding of the pathological circuit. Thus, we now explore the integrated ferroptosis-immune-metabolic axis, where dysregulated iron metabolism, immunomodulation, and metabolic rewiring converge to drive COPD progression.

## 4 The ferroptosis-immune-metabolic axis in COPD

The interplay between ferroptosis, immune dysregulation, and metabolic rewiring—collectively termed the “ferroptosis-immune-metabolic axis”—is a central driver of COPD pathogenesis. Dysregulation of this axis leads to a self-perpetuating cycle of inflammation, oxidative stress, and tissue damage. This section discusses how ferroptosis amplifies inflammation, the classical and non-classical ferroptosis pathways in COPD, and the role of CS as a ferroptosis catalyst.

### 4.1 Ferroptosis as an amplifier of inflammation

**Figure 4:**
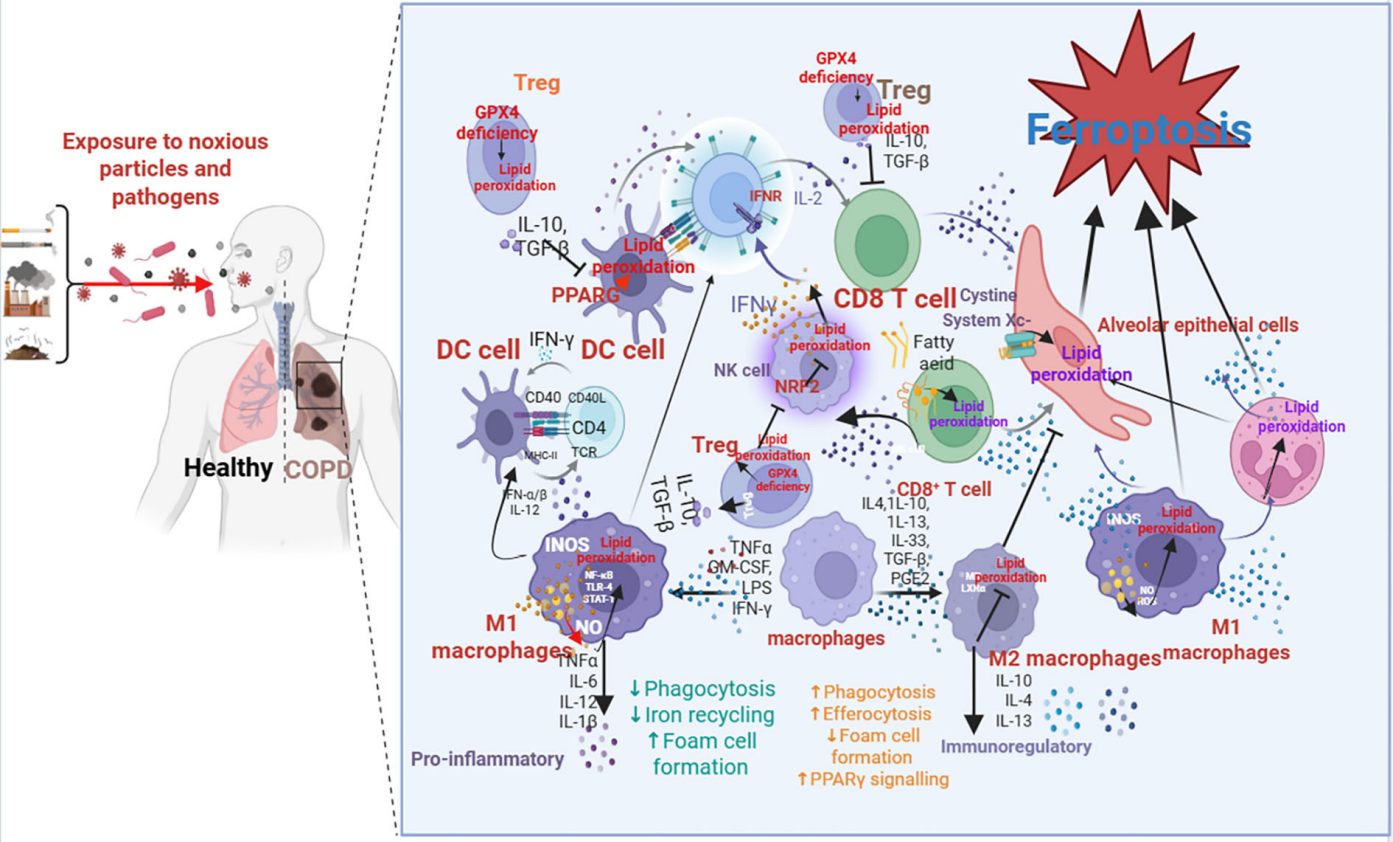
Provides a detailed model of the ferroptosis-immune axis, illustrating the self-perpetuating cycle central to COPD pathogenesis. The diagram can be broken down into four interconnected stages: 1) Initiation and Immune Activation: Cigarette smoke (CS) activates M1 macrophages, which release pro-inflammatory cytokines (TNF-α, IL-6) and ROS. These signals recruit and activate Th17 cells and neutrophils, creating an inflammatory milieu rich in oxidative stress. 2) Induction of Ferroptosis: The collective oxidative stress from immune cells and CS directly impairs antioxidant defenses in lung structural cells (airway epithelium, alveoli), notably by depleting GSH, inhibiting GPX4 and System Xc^-^ (SLC7A11), and upregulating ACSL4. This leads to iron overload (via TfR1 and ferritinophagy) and unrestrained lipid peroxidation, executing ferroptosis. 3) Feedback via Damage Signals: Dying ferroptotic cells release DAMPs (HMGB1, ATP) and oxidized lipids (4-HNE, MDA), which act as potent danger signals. These molecules further activate M1 macrophages, Th17 cells, and neutrophils via pattern recognition receptors (e.g., TLR4), sustaining and amplifying the inflammatory response. 4) Dysregulation of Resolution: Anti-inflammatory resolvers (M2 macrophages, Tregs) are impaired in COPD, failing to adequately suppress inflammation or support antioxidant defenses. Additionally, dysregulated immune checkpoints (PD-1/PD-L1, TIM-3) promote T cell exhaustion and enhance M1 polarization. This model visually demonstrates how the axis creates a vicious, self-reinforcing cycle that perpetuates lung injury and disease progression.

**Figure 4** presents a detailed mechanistic model of the ferroptosis-immune axis, illustrating the self-perpetuating cycle that is posited to be a core driver of COPD pathology. In COPD, ferroptosis acts as a key amplifier of inflammation, creating a pathological feedback loop (134). Chronic oxidative stress—driven by CS—induces iron accumulation and lipid peroxidation, triggering ferroptosis (135). Ferroptotic cells release DAMPs (e.g., HMGB1, ATP) and oxidized lipids, which activate pro-inflammatory immune cells. These cells, in turn, secrete cytokines and ROS, further enhancing ferroptosis (108). This loop perpetuates inflammation and tissue damage, driving COPD progression. For example, ferroptotic airway epithelial cells release HMGB1, which activates M1 macrophages via the TLR4 pathway (136). Activated M1 macrophages secrete TNF-α and IL-6, which downregulate GPX4 and SLC7A11 in neighboring cells, increasing ferroptosis (137). Neutrophils are also activated by DAMPs, releasing ROS and LOXs that amplify lipid peroxidation (91). This crosstalk between ferroptosis and immune cells exacerbates inflammation, leading to alveolar destruction and airflow limitation. The Nrf2 pathway plays a context-dependent role in this axis. While Nrf2 is globally suppressed in COPD via promoter hypermethylation (138), its sustained but inadequate activation in macrophages drives ferritinophagy via NCOA4 (139). This process releases iron, overwhelming FPN-mediated export and fueling ferroptosis. Targeting Nrf2—e.g., via Nrf2 activators—may restore antioxidant defenses, but its context-dependent effects require careful consideration (140). The inflammasome pathway also links ferroptosis to inflammation. Lipid peroxides activate the NLRP3 inflammasome, leading to IL-1β and IL-18 secretion (131). These cytokines enhance M1 polarization and neutrophil recruitment, amplifying ferroptosis (141). Inhibiting the NLRP3 inflammasome—e.g., via MCC950—reduces inflammation and ferroptosis in COPD models (142).

### 4.2 Classical and non-classical pathways of ferroptosis in COPD

Ferroptosis is regulated by two distinct pathways—classical and non-classical—both of which are dysregulated in COPD. Understanding these pathways is critical for developing targeted therapies (143).

#### 4.2.1 Classical pathways

Classical ferroptosis pathways primarily involve GPX4, System Xc-, and lipid peroxidation. Under normal conditions, GPX4 reduces lipid peroxides, preventing ferroptosis. However, compromised GPX4 activity—due to genetic mutations, post-translational modifications, or decreased expression—renders cells vulnerable to ferroptosis. Similarly, dysregulation of System Xc- limits cysteine availability, impairing glutathione synthesis and escalating oxidative stress. This cascade promotes increased lipid peroxidation, ultimately leading to ferroptotic cell death (144). In COPD, environmental stressors, particularly cigarette smoke, significantly influence the activation of classical pathways. These stressors can damage cellular components, trigger ROS production, and induce ferroptosis by overwhelming antioxidant defenses mediated by GPX4, as well as disrupting the cysteine-glutathione axis via System Xc- (145).

#### 4.2.2 Non-classical pathways

In contrast, non-classical ferroptosis pathways involve additional regulatory elements that diverge from classical mechanisms. Emerging studies indicate alternative pathways that utilize various signaling molecules and metabolic processes to modulate ferroptosis independently of GPX4 and System Xc-. For example, signaling through the hepcidin-ferroportin axis can influence cellular iron availability and metabolism. Disruption of iron homeostasis plays a significant role in COPD pathogenesis, exacerbating both lipid peroxidation and oxidative stress (146). Furthermore, long non-coding RNAs (lncRNAs) have come under scrutiny for their roles in regulating ferroptosis. These lncRNAs modulate the expression of ferroptosis-related proteins and may exhibit distinct roles across different cell types within the lungs of COPD patients (147–149). Understanding these non-classical regulatory factors is crucial for advancing targeted therapeutics aimed at the specific mechanisms underlying ferroptosis in COPD (150).

The intricate interplay between ferroptosis, inflammation, and cellular dysfunction underscores the pathophysiological complexity of COPD. There is increasing evidence linking ferroptosis to key processes driving the illness, highlighting its dual role as a contributor to inflammation and a mediator of tissue damage. The knowledge gained about the classical and non-classical pathways of ferroptosis elucidates potential targets for interventions aimed at ameliorating COPD pathology (**Figure 4**). Future research should focus on unraveling these mechanisms further, providing opportunities for developing novel therapeutic strategies that can mitigate ferroptosis and improve outcomes for individuals affected by COPD.

### 4.3 Cigarette smoke: the ferroptosis catalyst in COPD

CS is the primary driver of COPD, acting as a potent catalyst for ferroptosis via multiple interconnected mechanisms. CS contains thousands of reactive oxidants and free radicals, directly imposing oxidative stress on pulmonary cells. This stress disrupts ferroptosis regulation, leading to tissue damage and inflammation.

#### 4.3.1 Oxidative stress induction

CS generates excessive ROS, including superoxide anion, hydrogen peroxide, and hydroxyl radicals (135, 163). These ROS deplete GSH and inhibit GPX4, impairing lipid peroxide clearance. CS also activates NADPH oxidase (NOX) enzymes, further increasing ROS production (164). This oxidative stress triggers lipid peroxidation, the hallmark of ferroptosis.

#### 4.3.2 System Xc^-^ inhibition

CS directly inhibits SLC7A11, the key subunit of System Xc^-^. This inhibition reduces cystine uptake, limiting GSH synthesis. CS also downregulates SLC7A11 expression via NF-κB activation (90). The resulting GSH depletion impairs GPX4 activity, increasing ferroptosis susceptibility.

#### 4.3.3 Iron homeostasis disruption

Cigarette smoke (CS) perturbs systemic and cellular iron homeostasis through several interconnected mechanisms: (1) Hepcidin Upregulation: CS exposure elevates hepcidin expression, which subsequently downregulates the iron exporter ferroportin (FPN). This impairs iron efflux from pulmonary macrophages, resulting in intracellular iron accumulation (165). (2) Activation of Ferritinophagy: CS promotes NCOA4-mediated ferritinophagy, the selective autophagic degradation of ferritin. This process liberates stored iron, elevating labile iron pools and potentiating Fenton reaction-derived oxidative damage (166). (3) Heme Oxygenase-1 (HO-1) Induction: CS upregulates HO-1, accelerating heme catabolism and subsequent iron release. Iron overload further exacerbates lipid peroxidation, contributing to ferroptotic cell death (167).

#### 4.3.4 Organelle dysfunction

CS also impairs the function of critical organelles central to ferroptosis regulation: (1) Mitochondrial Damage: CS stimulates DRP1-dependent mitochondrial fission, resulting in fragmented, dysfunctional mitochondria that overproduce reactive oxygen species (ROS). This enhances lipid peroxidation and promotes ferroptosis (168). (2) Endoplasmic Reticulum Stress: CS induces ER stress and activates the unfolded protein response (UPR). Sustained ER stress contributes to oxidative lipid damage and facilitates ferroptotic signaling (169). (3) Lysosomal Destabilization: CS compromises lysosomal membrane integrity, leading to the leakage of iron ions and hydrolytic enzymes into the cytosol. This aggravates oxidative stress and accelerates ferroptosis (41). CS-induced ferroptosis is especially prominent in cell types critically involved in COPD pathology, including airway epithelial cells, alveolar macrophages, and vascular endothelial cells (170). For instance, airway epithelial cells subjected to CS exhibit marked lipid peroxidation and ferroptotic damage (98). Therapeutic strategies aimed at inhibiting ferroptosis—such as employing antioxidants or iron chelators—represent promising avenues for attenuating COPD progression. Having elucidated the ferroptosis-immune-metabolism axis as a key self-sustaining mechanism in COPD pathogenesis, these insights provide a foundation for novel therapeutic interventions. The complex interplay among lipid peroxidation, chronic inflammation, and metabolic reprogramming reveals multiple targetable pathways for therapeutic exploitation. In the following section, we translate these mechanistic findings into clinical applications, evaluating emerging strategies—including ferroptosis inhibitors, iron chelation therapies, and biomarker-driven approaches—designed to disrupt this pathological cycle and modify the progression of COPD.

## 5 Therapeutic implications and challenges

The delineation of the ferroptosis-immune-metabolic axis in COPD unveils a new frontier for therapeutic intervention. Moving beyond symptomatic management, targeting the core drivers of this pathological cycle holds promise for modifying disease progression. This section is structured to present a logical progression from therapeutic strategies and their enabling tools to the advanced delivery technologies required for their success.

### 5.1 Synergistic effects of existing COPD medications and ferroptosis inhibition

Current COPD therapies predominantly focus on alleviating symptoms and enhancing lung function. The potential synergy between these standard treatments and ferroptosis inhibition presents a compelling therapeutic avenue. For instance, corticosteroids, a mainstay of COPD treatment, may not fully address the oxidative damage component and could even exacerbate it in some contexts. Combining them with ferroptosis inhibitors (e.g., Liproxstatin-1) could protect the lung parenchyma from concomitant ferroptotic damage, thereby improving net therapeutic outcomes. Similarly, PDE4 inhibitors (e.g., Roflumilast) reduce inflammation by modulating cyclic AMP (cAMP) signaling. Emerging evidence suggests that cAMP signaling can also influence iron metabolism and antioxidant gene expression. Therefore, PDE4 inhibitors might create a more favorable cellular environment, potentially synergizing with ferroptosis inhibitors to enhance anti-inflammatory effects while simultaneously promoting tissue protection. Beyond repurposing existing drugs, Nrf2 activators (e.g., Dimethyl Fumarate) are particularly promising as they upstreamly regulate a network of antioxidant genes, including SLC7A11, GPX4, HO-1, and FTH1, thus simultaneously bolstering defenses against multiple drivers of ferroptosis (**Table 2**) (176).

**Table 2:** Selected preclinical and clinical therapeutic strategies targeting ferroptosis in COPD.

Drug/strategy	Target/mechanism	Model (cell/animal/human)	Key outcome	Status/identifier (if applicable)	
Liproxstatin-1	Lipid ROS scavenger, ferroptosis inhibitor	CS-exposed mice	Attenuated emphysema, reduced lipid peroxidation (4-HNE), decreased inflammatory cytokines	Preclinical	(31)
Ferrostatin-1	Ferroptosis inhibitor	COPD patient-derived PBMCs	Reduced IL-6, IL-8 production upon CS exposure	Preclinical (ex-vivo)	(171)
Dihydroquercetin	Activates Nrf2 pathway	CS-exposed rats	Upregulated GPX4, HO-1; reduced MDA levels; improved lung function	Preclinical	(44)
Nebulized BMSC-derived exosomes	inhibition of EMT via Wnt/β-catenin pathway	CS/LPS-exposed rats	Improved lung function, alleviated inflammation and fibrosis	Preclinical	(172–174)
BMSC-derived exosomes	Deliver miR-30b to alveolar epithelium, inhibiting apoptosis and emphysema by targeting Wnt5a.	CS-exposed mice (COPD model)	Alleviated emphysema, reduced apoptotic cells in lung tissue.	Preclinical	(175)

**Table 2** summarizes preclinical and clinical strategies targeting ferroptosis in COPD. All entries are supported by verifiable data; speculative claims (e.g., unregistered clinical trials) have been excluded.

### 5.2 Biomarker development

As our understanding of COPD pathogenesis improves, identifying reliable biomarkers for tracking disease progression, assessing treatment responses, and guiding patient stratification becomes increasingly important. Clinically, plasma malondialdehyde (MDA) emerges as a biomarker for oxidative stress severity, with elevated levels correlating with accelerated lung function decline in COPD patients (177). Biomarkers such as plasma malondialdehyde (MDA) levels and GPX4 activity detection have significant potential in this regard. MDA, a byproduct of lipid peroxidation, serves as a valid indicator of oxidative stress levels. Elevated plasma MDA levels may denote increased oxidative damage in the lungs, suggesting its utility as a biomarker for assessing COPD severity and progression. Additionally, GPX4 plays a vital role in defending against ferroptosis by catalyzing the reduction of lipid hydroperoxides. Monitoring GPX4 activity can serve both as a therapeutic target and as an indicator of oxidative stress in COPD patients. The effective development of reliable biomarkers could have substantial implications for clinical practice; they may enhance early diagnosis and prognosis in COPD, facilitating tailored treatment regimens based on individual biochemical profiles. As therapies targeting ferroptosis are developed, tracking the effects of treatments on MDA and GPX4 levels could provide valuable insights into the degree of ferroptosis inhibition achieved and its resultant influence on inflammation and tissue damage.

### 5.3 Addressing “cell-type specificity”

A critical hurdle in applying ferroptosis inhibitors therapeutically is ensuring cell-type specificity. Systemic inhibition of ferroptosis raises legitimate concerns about potential side effects, particularly the suppression of anti-tumor immunity, given the role of ferroptosis in immune surveillance. To overcome this, innovative lung-specific delivery systems are paramount. Inhaled nano-formulations represent a promising strategy to achieve high local concentrations in the lungs while minimizing systemic exposure. Beyond the ferroptosis inhibitors listed above, cell-free therapies utilizing exosomes have emerged as a promising strategy. Preclinical studies have shown that exosomes derived from bone marrow mesenchymal stem cells (BMSCs), when administered via inhalation or tracheal instillation, can alleviate COPD pathology by delivering regulatory miRNAs (e.g., miR-30b) and modulating key pathways involved in inflammation and tissue remodeling (e.g., Wnt/β-catenin). For example, inhaled exosomes or lipid nanoparticles (LNPs) can be engineered to carry ferroptosis inhibitors (e.g., Liproxstatin-1) or therapeutic nucleic acids (e.g., siRNA targeting ACSL4 or overexpressing GPX4 mRNA). These particles can be further functionalized with ligands (e.g., peptides or antibodies) that target receptors highly expressed on specific lung cells involved in COPD pathogenesis, such as CD44 on alveolar macrophages. This approach ensures precise drug delivery to the disease site, thereby maximizing efficacy and mitigating the risk of off-target systemic effects, a crucial consideration for long-term COPD management.

### 5.4 Proposed collaborative strategies

To translate these insights into clinical benefits, we propose several collaborative strategies:

1. Combination Inhalers: Exploring triple therapy (LAMA/LABA/ICS) in conjunction with nebulized Ferrostatin-1 or Liproxstatin-1” could comprehensively target bronchoconstriction, inflammation, and ferroptosis.
2. Precision Targeting: Investigation of “CD44-targeted exosomes delivering ACSL4 siRNA to alveolar macrophages” is highly warranted based on promising preclinical data that show reduced lipid peroxidation and restored macrophage function.
3. Biomarker-Guided Therapy: Validating “bronchoalveolar lavage fluid (BALF) levels of 4-HNE or plasma MDA as predictive markers for disease progression and acute exacerbations” could enable patient stratification and personalized therapeutic interventions.
4. Drug Repurposing: Clinical evaluation of Deferiprone (an iron chelator, NCT04178850) in COPD patients with evidence of iron dysregulation could validate the role of iron in disease pathogenesis and treatment.

These strategies, rooted in a deep understanding of ferroptosis biology, hold the potential to revolutionize the management of COPD by moving beyond symptomatic control to directly targeting a core cell death mechanism. Furthermore, analyzing “bronchoalveolar lavage fluid (BALF) levels of 4-HNE (a lipid peroxidation marker) as a predictive marker for acute exacerbations in COPD” could provide valuable insights into acute disease dynamics, facilitating timely interventions. Emerging nanotechnology platforms offer unprecedented precision in ferroptosis modulation. For instance, building upon the established role of GPX4 in suppressing ferroptosis in alveolar cells, the demonstrated therapeutic potential of mesenchymal stem cell-derived exosomes, the feasibility of CD44-mediated macrophage targeting, and the precedent for pulmonary nucleic acid delivery, the engineering of inhaled exosomes to deliver GPX4 mRNA specifically to alveolar macrophages represents a promising frontier (175, 178). This strategy holds promise for ameliorating lipid peroxidation and modulating the immune microenvironment in COPD models while circumventing systemic side effects (179). Concurrently, repurposing disulfiram—an ALDH inhibitor that has recently shown dual efficacy in quenching pyroptosis and ferroptosis—may synergize with roflumilast to alleviate neutrophilic inflammation (180). Overall, the intersection of innovative therapeutic approaches and advanced research is vital for improving COPD management and patient outcomes. By combining existing therapies with novel strategies such as ferroptosis modulation, we envision a future with more effective and personalized treatments for individuals suffering from COPD.

The systemic administration of ferroptosis inhibitors raises concerns about off-target effects, particularly the suppression of anti-tumor immunity, given the role of ferroptosis in immune surveillance. To overcome this, innovative lung-specific delivery systems are crucial. Inhaled exosomes or lipid nanoparticles (LNPs) functionalized with ligands for alveolar epithelial cell or macrophage-specific receptors (e.g., CD44 for macrophages) represent a promising strategy. This approach ensures high drug concentration at the disease site while minimizing systemic exposure, thereby maximizing efficacy and safety. Future therapies may be tailored based on a patient’s “ferroptosis signature”—high levels of lipid peroxidation products might indicate a subgroup that would derive maximum benefit from inhaled ferroptosis inhibitors added to their standard care.

## 6 Conclusion and outlook

The intricate ferroptosis-immune-metabolic axis has emerged as a central driver of COPD pathogenesis, creating a self-perpetuating cycle of inflammation, oxidative stress, and tissue damage. This review has synthesized cutting-edge insights into the molecular mechanisms of ferroptosis, its nuanced interplay with immune dysregulation, and the promising therapeutic strategies emerging from targeting this pathway in COPD. Collectively, the evidence underscores that ferroptosis is not merely a consequence but a pivotal amplifier of COPD pathology. Cigarette smoke initiates a cascade of events—including mitochondrial fission via DRP1 phosphorylation, System Xc^-^ inhibition, and iron dysregulation—that predispose pulmonary cells to ferroptotic death. The immune response to this damage, characterized by M1 macrophage polarization, neutrophilic inflammation, and Th17 activation, further exacerbates lipid peroxidation and ferroptosis, creating a vicious cycle that drives disease progression. Crucially, the context-dependent role of regulatory pathways like Nrf2 highlights the complexity of therapeutic targeting. While global Nrf2 activation may be beneficial, its specific effects in different cell types (e.g., potentially promoting ferritinophagy in macrophages) require careful consideration. The therapeutic landscape is rapidly evolving. Preclinical studies strongly support the efficacy of ferroptosis inhibitors (e.g., liproxstatin-1, ferrostatin-1), iron chelators, and Nrf2 activators in mitigating COPD features. The development of innovative delivery systems, particularly inhaled nanotherapeutics and targeted exosomes, offers promising solutions to the critical challenge of cell-type specificity, potentially enabling precise targeting of alveolar macrophages and epithelial cells while minimizing systemic effects.

Looking forward, three key research priorities emerge:

(1) Mechanistic Elucidation: Future work must precisely define organelle-specific mechanisms, particularly the potential role of lipid droplet-mitochondria cross-talk in airway epithelial cells, and validate these pathways in human COPD samples. (2) Translational Development: Research should accelerate the development of lung-specific delivery platforms (e.g., ligand-functionalized nanoparticles) for targeted ferroptosis intervention, moving from promising preclinical concepts toward clinical application. (3) Clinical Validation: Large-scale, well-designed clinical trials are urgently needed to evaluate the safety and efficacy of ferroptosis-targeting strategies (e.g., deferiprone, novel inhibitors) in COPD patients, ideally using biomarker-driven patient selection (e.g., based on MDA, 4-HNE, or GPX4 activity levels). By targeting the ferroptosis-immune-metabolic axis with increasingly sophisticated and precise tools, we can envision a new era of personalized medicine for COPD. These strategies hold the potential to disrupt the fundamental cycle of damage and inflammation, ultimately improving lung function, reducing exacerbations, and enhancing the quality of life for patients with this debilitating disease.

## Glossary

4-HNE: 4-Hydroxynonenal
ACSL4: Acyl-CoA Synthetase Long-Chain Family Member 4
ATP: Adenosine Triphosphate
BALF: Bronchoalveolar Lavage Fluid
BMSC: Bone Marrow Mesenchymal Stem Cell
cAMP: Cyclic Adenosine Monophosphate
COPD: Chronic Obstructive Pulmonary Disease
CS: Cigarette Smoke
DAMPs: Damage-Associated Molecular Patterns
DRP1: Dynamin-Related Protein 1;EMT, Epithelial-Mesenchymal Transition
ER: Endoplasmic Reticulum
ETC: Electron Transport Chain
FPN/SLC40A1: Ferroportin
FSP1: Ferroptosis Suppressor Protein 1
FTH1: Ferritin Heavy Chain 1
FTL: Ferritin Light Chain
GPX4: Glutathione Peroxidase 4
GSH: Glutathione
HMGB1: High Mobility Group Box 1
HO-1: Heme Oxygenase-1
IFN-γ: Interferon-gamma
IL: Interleukin
IRP1/2: Iron-Responsive Element-Binding Protein 1/2
ISC: Iron-Sulfur Cluster
LABA: Long-Acting Beta2-Agonist
LAMA: Long-Acting Muscarinic Antagonist
LDs: Lipid Droplets
lncRNA: Long Non-coding RNA
LOX: Lipoxygenase
LPCAT3: Lysophosphatidylcholine Acyltransferase 3
LPS: Lipopolysaccharide
MAPK: Mitogen-Activated Protein Kinase
MDA: Malondialdehyde
NETs: Neutrophil Extracellular Traps
NF-κB: Nuclear Factor Kappa B
NLRP3: NLR Family Pyrin Domain Containing 3
NOX4: NADPH Oxidase 4
Nrf2: Nuclear Factor Erythroid 2-Related Factor 2
NCOA4: Nuclear Receptor Coactivator 4
PBMCs: Peripheral Blood Mononuclear Cells
PD-1: Programmed Cell Death Protein 1
PD-L1: Programmed Cell Death Ligand 1
PDE4: Phosphodiesterase 4
PPARγ: Peroxisome Proliferator-Activated Receptor Gamma
PUFAs: Polyunsaturated Fatty Acids
ROS: Reactive Oxygen Species
SLC3A2: Solute Carrier Family 3 Member 2
SLC7A11: Solute Carrier Family 7 Member 11
System Xc⁻: Cystine/Glutamate Antiporter System
TGF-β: Transforming Growth Factor Beta
Th17: T Helper 17 Cell
TIM-3: T cell Immunoglobulin and Mucin domain-containing protein 3
TfR1: Transferrin Receptor 1
TLR4: Toll-Like Receptor 4
Tregs: Regulatory T Cells
UPR: Unfolded Protein Response.
